# An Evaluation of the Sensitivity and Specificity of Three COVID-19 Rapid Immunochromatographic Test Kits Compared to Real-Time Reverse Transcriptase-Polymerase Chain Reaction (rRT-PCR) Among Clinical Samples

**DOI:** 10.7759/cureus.63294

**Published:** 2024-06-27

**Authors:** S Bani, Ebenezer K Amakye, Shanti Akomea, Eric Ny Nyarko, Derrick Dodoo, Clement Aidoo, Magdalene Fynn-Buadu, Monica Adom, Fathea Bani, Christian Obirikorang

**Affiliations:** 1 Department of Biomedical Laboratory Sciences, School of Allied Health Sciences, University for Development Studies, Tamale, GHA; 2 Chemical Pathology, University of Ghana Medical School, Accra, GHA; 3 Department of Medical Laboratory Sciences, Baldwin University College, Accra, GHA; 4 Department of Medical Laboratory Sciences, Accra Technical University, Accra, GHA; 5 Department of Medical Laboratory Sciences, Princess Marie Louise Children’s Hospital, Ghana Health Service, Accra, GHA; 6 Department of Medicine, School of Allied Health Sciences, University for Development Studies, Tamale, GHA; 7 Global Health and Infectious Diseases Group, Kumasi Centre for Collaborative Research, Department of Molecular Medicine, School of Medicine and Dentistry, Kwame Nkrumah University of Science and Technology, Kumasi, GHA

**Keywords:** sormas, rapid antigen test kits, polymerase chain reaction (pcr), sars-cov-2, covid-19

## Abstract

Background and objective

The coronavirus disease 2019 (COVID-19) pandemic has imposed a significant burden on healthcare systems worldwide. This highlights the need for simple, rapid, and affordable diagnostic tests that can serve as alternatives to the existing costly and demanding polymerase chain reaction (PCR) assay, especially in resource-limited countries like Ghana. In light of this, we aimed to assess the diagnostic efficacy of three COVID-19 rapid immunochromatographic antigen test kits vs. real-time reverse transcriptase-PCR (rRT-PCR).

Methods

This study evaluated the sensitivity and specificity of three COVID-19 rapid immunochromatographic antigen test kits: DG Rapid, SD Rapid, and SS Rapid. They were compared with the gold standard RT-PCR for the detection of severe acute respiratory syndrome coronavirus 2 (SARS-CoV-2) nucleocapsid antigen in 75 randomly selected archived nasopharyngeal samples.

Results

Of the 75 samples tested, 38 (50.7%) were positive and 37 (49.3%) were negative for SARS-CoV-2 RNA by rRT-PCR assay. No false positives were recorded. On the other hand, the DG Rapid kit detected 30 (78.9%) true positives and eight (21.1%) false negatives. SD Rapid kit detected 28 (73.7%) true positives and 10 (26.3%) false negatives, while the SS Rapid kit detected 19 (50.0%) true positives and 19 (50.0%) false negatives. While the specificity of each test kit was 100% (95% CI), the sensitivity of the DG Rapid, SD Rapid, and SS Rapid kits was 79%, 74%, and 50% (95% CI), respectively. Higher sensitivities were recorded among samples with cycle threshold (Ct) values <29.99 for each kit. Also, the DG Rapid kit demonstrated 79% excellent agreement with rRT-PCR, while the SD Rapid and SS Rapid kits demonstrated good agreement with rRT-PCR with 73% and 50% Cohen’s kappa values, respectively.

Conclusions

Based on our findings, DG Rapid and SD Rapid kits are reliable alternatives to rRT-PCR for the detection of SARS-CoV-2 infection, especially in resource-limited settings like Ghana.

## Introduction

The World Health Organization (WHO) declared the outbreak of coronavirus disease 2019 (COVID-19), caused by severe acute respiratory syndrome coronavirus 2 (SARS-CoV-2), a global pandemic on March 12, 2020 [1]. COVID-19 originated in the Hubei province of Wuhan, China, where a cluster of cases of pneumonia with unknown etiology was reported in early December 2019 [2,3]. The novel coronavirus was identified as the etiological agent, and the disease subsequently spread rapidly to other cities in China and other countries worldwide [4]. Globally, 632,895,390 confirmed cases and 6,596,852 related deaths were reported to WHO as of November 7, 2022 [5]. In Ghana, the first confirmed case was recorded on March 12, 2020 [6], and the number of confirmed cases reported to WHO from all regions of the country stood at 170,972, with 1,461 deaths as of November 7, 2022 [5].

At the height of the pandemic, several real-time reverse transcriptase-polymerase chain reaction (rRT-PCR) laboratories were set up, and other existing laboratories were revamped to enhance the preparedness of the country in terms of testing suspected cases [7]. However, rRT-PCR is costly, time-consuming, and requires specially trained personnel to execute [8]. The rapid immunochromatographic antigen test may be a cost-effective substitute for this task and may be used directly and instantly, offering results within a few minutes. This enables speedy decision-making, significantly affecting clinical management outcomes [9]. This study aimed to assess the diagnostic efficacy of three COVID-19 rapid antigen test kits (RDT) - the DG Rapid RDT (DG Rapid), SD Rapid RDT (SD Rapid), and SS Rapid RDT (SS Rapid) - that rely on the immunochromatographic technique.

The kits were selected based on their approval by the Ghana Food and Drugs Authority (FDA) and their availability on the Ghanaian market, as the reliance on such RDTs continues to grow. The specific objectives were to determine and compare the sensitivity, specificity, and predictive values of the three antigen test kits to known rRT-PCR-tested nasopharyngeal samples. Other objectives included assessing the test kits to determine if their sensitivity, specificity, and predictive values aligned with the manufacturer's claims; comparing the sensitivity, specificity, and predictive values of the test kits with the known cycle threshold (Ct) values obtained from rRT-PCR tested samples; and evaluating the overall agreement of these test kits against the gold standard rRT-PCR.

## Materials and methods

Study design, setting, and sample selection criteria

We conducted an experimental retrospective study of archived COVID-19 rRT-PCR samples. Ethical approval was sought and obtained from the Institutional Review Board, University for Development Studies (UDS/IRB/115/22). Three packets (75 pcs) each of DG Rapid, SD Rapid, and SS Rapid, which rely on the immunochromatographic technique, were randomly acquired from the Ghanaian market. The kits were also selected based on their approval by the FDA. The study was conducted at a Food and Drugs Authority/Health Facilities Regulatory Agency (FDA/HeFRA)-registered SARS-CoV-2 rRT-PCR testing facility in the Greater Accra Region of Ghana, between June 2022 and July 2022. The inclusion criteria for this study were as follows: clinical samples that were previously tested for COVID-19 using the rRT-PCR technique. However, insufficient samples, inadequately labeled samples, or samples with other sample quality issues were excluded from the study. There were no restrictions in terms of age, gender, or ethnicity.

Sample analysis

Seventy-five (75) frozen archived nasopharyngeal samples (38 COVID-19 rRT-PCR-positive and 37 rRT-PCR-negative) were obtained for the evaluation of the three brands of immunochromatographic RDT kits. The archived samples were selected using a flow chart (see the image in Appendices). It is worth noting that these samples were retested using rRT-PCR (the reference test: S0) before the immunochromatographic assays (the index test: S1). The three immunochromatographic antigen kits (i.e., DG Rapid, SD Rapid, and SS Rapid) consist of qualitative membrane-based immunoassays and detect SARS-CoV-2 nucleocapsid antigens. All assays were performed by a single operator for each brand of test kit, with two blind operators assessing the results. In case of discrepancies, a third operator was consulted, especially for faint test lines. All tests were performed according to the manufacturer’s instructions [10-12].

Data collection and analysis

The results generated were entered into Microsoft Excel-365 and analyzed using IBM SPSS Statistics version 23 (IBM Corp., Armonk, NY).

## Results

The detection rate of the three brands of COVID-19 rapid immunochromatographic test (ICT) kits

Table 1 shows the results related to some quality characteristics for the three different test kits: DG Rapid, SD Rapid, and SS Rapid. These results indicate that the DG Rapid and SD Rapid groups had a higher proportion of true positives (TPs) and a lower proportion of false negatives (FNs) than the SS Rapid group. However, all test kits recorded 100% true negatives (TN) and zero cases of false positives (FPs), suggesting high specificity for all three kits.

**Table 1:** Results indicating the detection rate of the three brands of COVID-19 rapid ICT kits COVID-19: coronavirus disease 2019; ICT: immunochromatography test

Test kits	True positive, n (%)	True negative, n (%)	False negative, n (%)	False positive, n (%)
DG Rapid	30 (78.9)	37 (100.0)	8 (21.1)	0
SD Rapid	28 (73.7)	37 (100.0)	10 (26.3)	0
SS Rapid	19 (50.0)	37 (100.0)	19 (50.0)	0

Diagnostic performance of COVID-19 rapid ICT kits

Table 2 presents the sensitivity, specificity, positive predictive value (PPV), negative predictive value (NPV), and overall accuracy of the three different ICT kits. The sensitivity of DG Rapid and SD Rapid was relatively high, with values of 78.9% and 73.7%, respectively, while the sensitivity of SS Rapid was lower at 50.0%. All three kits demonstrated 100.0% specificity and 100% PPV. The NPVs of DG Rapid, SD Rapid, and SS Rapid were 82.2%, 78.7%, and 66.1%, respectively. The overall accuracy was highest for DG Rapid at 89.3%, followed by SD Rapid at 86.7%, and SS Rapid at 74.7%. These results suggest that DG Rapid and SD Rapid may be more effective than SS Rapid for detecting SARS-CoV-2 RNA.

**Table 2:** Sensitivity, specificity, PPV, NPV, and overall accuracy of the ICT kits ICT: immunochromatography test; NPV: negative predictive value; PPV: positive predictive value

Test kits	Sensitivity, %	Specificity, %	PPV, %	NPV, %	Overall accuracy, %
DG Rapid	78.9	100.0	100.0	82.2	89.3
SD Rapid	73.7	100.0	100.0	78.7	86.7
SS Rapid	50.0	100.0	100.0	66.1	74.7

Comparison of overall accuracy and Cohen’s kappa coefficient between the manufacturers and the operators

Table 3 presents a comparison of the overall accuracy and Cohen's kappa coefficient regarding COVID-19 rapid ICT kits between the manufacturers and the operators. Overall accuracy varies with disease prevalence, and it may be misleading if the study population differs from the actual population. Cohen’s kappa coefficient was used as an alternative to compare the prediction performance of the ICT kits to rRT-PCR with kappa values indicating agreement levels; >75% implying excellent/total agreement, 40-75% good agreement, and <40% poor agreement with rRT-PCR. When the manufacturers' and operators' analyses were compared, the overall accuracy and Cohen's kappa coefficient of the operators' analyses were lower than those of the manufacturers. Nonetheless, the results still indicate that the DG Rapid kit is the most reliable ICT kit in both manufacturers' and operators' analysis.

**Table 3:** Comparison of overall accuracy and Cohen’s kappa coefficient of ICT kits between the manufacturers and the operators ICT: immunochromatography test

Test Kits	Manufacturers’ overall accuracy, %	Manufacturers’ Cohen’s kappa, %	Operators’ overall accuracy, %	Operators’ Cohen’s kappa, %
DG Rapid	98.8	97.3	89.3	78.7
SD Rapid	98.4	89.1	86.7	73.4
SS Rapid	99.2	96.5	74.7	49.7

Diagnostic performance of the operators' ICT kits based on rRT-PCR Ct value categories

Table 4 demonstrates the performance of the operators' ICT kits based on rRT-PCR Ct Values. To determine the diagnostic performance characteristics of the test kits in comparison with viral load, the Ct values were classified into the following categories to assess the threshold at which the test kits would have the best performance: 19.00 - 29.99, 30.00 - 34.99, and >35.00. The results showed that the DG Rapid and SD Rapid kits had higher sensitivity and Cohen's kappa coefficient than the SS Rapid kit in detecting SARS-CoV-2 across all Ct value categories. However, all three kits showed high specificity and PPV in all Ct value categories. Specifically, Cohen's kappa coefficient indicated substantial or excellent agreement between the ICT kits and rRT-PCR results at lower Ct values (19.00 - 29.99 and 30.00 - 34.99) for the DG Rapid and SD Rapid kits, but only good agreement for the SS Rapid kit. However, all three kits had poor agreement with rRT-PCR for Ct values >35.00.

**Table 4:** Diagnostic performance of the operators' ICT kits based On rRT-PCR Ct value categories Ct: cycle threshold; ICT: immunochromatography test; NPV: negative predictive value; PPV: positive predictive value; rRT-PCR: real-time reverse transcriptase-polymerase chain reaction

Test kits (N=75)	rRT-PCR Ct values	Sensitivity, %		Specificity, %		PPV, %	NPV, %	Overall accuracy, %	Cohen’s kappa coefficient, %
DG Rapid (n=24)	19.00 - 29.99	91.7	100		100		94.9	96.7	93.0
DG Rapid (n=7)	30.00 - 34.99	85.7	100		100		97.4	97.7	91.0
DG Rapid (n=7)	>35.00	28.6	100		100		88.1	88.6	40.2
SD Rapid (n=24)	19.00 - 29.99	88.9	100		100		92.5	95.3	90.2
SD Rapid (n=7)	30.00 - 34.99	71.4	100		100		94.9	95.5	80.8
SD Rapid (n=7)	>35.00	28.6	100		100		88.1	88.6	40.2
SS Rapid (n=24)	19.00 - 29.99	58.3	100		100		78.7	83.6	62.9
SS Rapid (n=7)	30.00 - 34.99	57.1	100		100		92.5	93.2	69.2
SS Rapid (n=7)	>35.00	14.3	100		100		86.0	86.4	21.9

Comparison of the overall accuracy and Cohen’s kappa coefficient based on rRT-PCR Ct values for operators’ ICT kits

Table 5 shows the comparison between the test kits in terms of various rRT-PCR Ct value categories concerning overall accuracy and Cohen’s kappa coefficient to ascertain if the Ct values affected any of these diagnostic performance indicators. The results showed that the DG Rapid and SD Rapid kits had higher overall accuracy and Cohen's kappa coefficient than the SS Rapid kit across all Ct value categories. The DG Rapid kit had the highest overall accuracy and Cohen's kappa coefficient for Ct values of 19.00 - 29.99 and 30.00 - 34.99, while the SD Rapid kit had the second highest overall accuracy and Cohen's kappa coefficient for the same ranges. The SS Rapid kit had the lowest overall accuracy and Cohen's kappa coefficient across all Ct value categories. However, all three kits recorded the lowest overall accuracy and Cohen's kappa coefficient for Ct values >35.00.

**Table 5:** Comparison of overall accuracy and Cohen’s kappa coefficient of operators’ ICT kits based on rRT-PCR Ct value categories Ct: cycle threshold; ICT: immunochromatography test; rRT-PCR: real-time reverse transcriptase-polymerase chain reaction

Test kits (N=75)	rRT-PCR Ct values	Overall accuracy, %	Cohen’s kappa coefficient, %
DG Rapid (n=24)	19.00 - 29.99	96.7	93.0
DG Rapid (n=7)	30.00 - 34.99	97.7	91.0
DG Rapid (n=7)	>35.00	88.6	40.2
SD Rapid (n=24)	19.00 - 24.99	95.3	90.2
SD Rapid (n=7)	30.00 - 34.99	95.5	80.8
SD Rapid (n=7)	>35.00	88.6	40.2
SS Rapid (n=24)	19.00 - 24.99	83.6	62.9
SS Rapid (n=7)	30.00 - 34.99	93.2	69.2
SS Rapid (n=7)	>35.00	86.4	21.9

## Discussion

This study evaluated the diagnostic performance characteristics of these brands of COVID-19 ICT test kits available in the Ghanaian market, namely, DG Rapid, SD Rapid, and SS Rapid. Rapid antigen tests offer several advantages, including affordability, faster turnaround time, and the ability to diagnose patients at their point of care. These advantages are essential and critical, especially in resource-limited settings, where rRT-PCR testing may not be readily available. This study demonstrated that DG Rapid and SD Rapid antigen test kits performed relatively better in detecting SARS-CoV-2 compared to SS Rapid.

The findings for DG Rapid and SD Rapid are consistent with previous studies that have reported the effectiveness of rapid antigen tests in detecting COVID-19. These studies have shown a relatively lower sensitivity with SARS-CoV-2 antigen rapid diagnostic test kits compared to the clinical reference standard, i.e., rRT-PCR. A recent systematic review and meta-analysis by Brümmer et al. [13] that evaluated the accuracy of commercially available SARS-CoV-2 antigen rapid diagnostic test kits revealed a pooled sensitivity of 71.2%. A sensitivity of 70% and 59% (95% CI) was observed among 262 study participants in Uganda and Cameroon, respectively, using the SD Rapid RDT [14,15]. In Cameroon, the RDTs’ sensitivity of 59% (95% CI) increased to 69% (95% CI) when only symptomatic participants were considered. Another study conducted at a teaching hospital in northern Ghana involving 193 participants reported the sensitivity of the SD Rapid RDT as 64% (95% CI) [16].

Our study, however, found a sensitivity of 74% (95% CI) for SD Rapid RDT, which is comparable to what was observed in Uganda and Cameroon. Moreover, among the three brands, DG Rapid demonstrated a higher sensitivity of 79% (95% CI), followed by SD Rapid with 74% (95% CI). However, the SS Rapid RDT demonstrated a lower sensitivity of 50% (95% CI) compared to the other two brands. This lower sensitivity for SS Rapid may limit its utility as a standalone diagnostic tool, as it may lead to false-negative results. False-negative results may result in a delay in diagnosis, thereby increasing the risk of virus transmission. It is noteworthy that these values of sensitivity observed in our study are much lower when compared to those reported by the manufacturers (DG Rapid, 2022; SD Rapid, 2020; SS Rapid, 2021)[ 17]. This could be attributed to factors such as variations in the batch of ICT kits used, variations in the concentration of extracted antigens, differences in processing techniques, and variations in the storage conditions of the kits, especially in the market [18].

In this study, the specificity was 100% (95% CI), which aligns with the 100% (95% CI) value claimed by the manufacturers but is higher than the 92% (95% CI) documented in the studies conducted in both Uganda and Cameroon. The lower specificity values in these other studies were linked to cross-reacting antibodies from previous infections or variations in environmental testing temperatures (24 - 37 °C) in the general wards and the COVID-19 isolation center where the tests were carried out [16]. The high specificity exhibited by all three test kits in our study is an important attribute as it ensures that individuals without the virus are correctly identified, reducing the risk of false-positive results. False positives can lead to unwarranted quarantine, isolation, and treatment, with significant social and economic consequences.

Regarding overall accuracy, this study reported values of 89% (DG Rapid), 87% (SD Rapid), and 75% (SS Rapid) at 95% CI for the three SARS-CoV-2 ICT kits, which are lower than the manufacturers' claims of 99%, 98%, and 99%, respectively. However, it is essential to recognize that overall accuracy can vary with disease prevalence, making it less reliable as a single summary measure of a test's validity. The prevalence-dependent nature of overall accuracy introduces challenges, leading to warnings against its use. Estimates of overall accuracy can be misleading when obtained from populations with significantly different disease prevalence from the target population in whom the test is intended for application [19].

To assess the agreement between the ICT kits and rRT-PCR, Cohen's kappa coefficient was employed. Specifically, it was used to determine the level of agreement between the performance of the ICT kits and rRT-PCR. In our study, only DG Rapid showed excellent agreement, while SD Rapid and SS Rapid exhibited good agreement compared to rRT-PCR. This could be because DG Rapid demonstrated a higher sensitivity of 79% and a higher NPV of 82% compared to the other brands. Other performance indicators, specifically, specificity and PPV, were similar (100%) for all three brands and thus did not have an impact on the kappa value calculation. In a study from India, Cohen's kappa calculated for SD Rapid and rRT-PCR showed a good agreement, with a Cohen's kappa of 64.4% [20]. Another study from Ethiopia found that a SARS-CoV-2 antigen rapid test kit and rRT-PCR had a kappa value of agreement of 73.5%, indicating good agreement between the two tests [21]. While we could not find specific studies on DG and SS Rapid kits, the findings of the studies from both India and Ethiopia suggest a consistent trend of good agreement between SARS-CoV-2 antigen rapid test kits, such as SD Rapid, and rRT-PCR.

By convention, a lower Ct value signifies a higher viral load, while a higher Ct value suggests a lower viral load. All three brands demonstrated better detection limits for higher viral load (Ct values ≤29.99), which is often the case in the pre-symptomatic phase (one to three days before the onset of symptoms) and the early symptomatic phase (during the first five to seven days of illness) of SARS-CoV-2 infection [22]. Conversely, they displayed less favorable detection limits for Ct values >35.00. Consequently, the DG Rapid ICT kits exhibit a lower detection threshold when Ct values exceed 35.00, despite their excellent agreement with rRT-PCR. 

The findings of this study have significant implications for public health, policy, and clinical practice. These kits can enhance testing capacity in resource-limited settings, enabling quicker identification and isolation of positive COVID-19 cases, which is crucial in controlling the spread of COVID-19. Policymakers can use these findings to allocate resources more effectively, and clinicians and medical technologists can incorporate rapid tests into their diagnostic protocols, especially when rRT-PCR is not feasible.

Limitations of the study

This study has a few limitations. Primarily, it is essential to acknowledge that our study utilized frozen archived nasopharyngeal samples collected from the Greater Accra Region between 2020 and 2022, which may not represent the current situation accurately. Moreover, the performance of these antigen test kits can be influenced by factors such as viral load and the type of specimen used for testing.

## Conclusions

Our findings indicate that the DG Rapid and SD Rapid kits can be reliable substitutes for rRT-PCR in detecting SARS-CoV-2 infection, particularly in areas with scarce resources and limited access to PCR testing. The results also suggest that these test kits exhibit a high level of sensitivity in samples with Ct values lower than 29.99, indicating that they would perform well in patients with a high viral load (Ct values ≤29.99). These findings endorse the use of rapid antigen tests as an alternative diagnostic tool for COVID-19. Based on these findings, we recommend a further study with a larger, fresh sample size on the other available COVID-19 RDT kits in the Ghanaian market, as rRT-PCR testing is expensive and not widely available. We also recommended that these test kits (DG Rapid, SD Rapid, and SS Rapid), which showed reliable sensitivity and specificity, be utilized extensively, especially where rRT-PCR is not readily accessible or affordable. These tests can provide quick results, aiding in timely decision-making and patient management, thereby improving clinical practice and health delivery systems in resource-limited settings like Ghana.

## Figures and Tables

**Figure 1:**
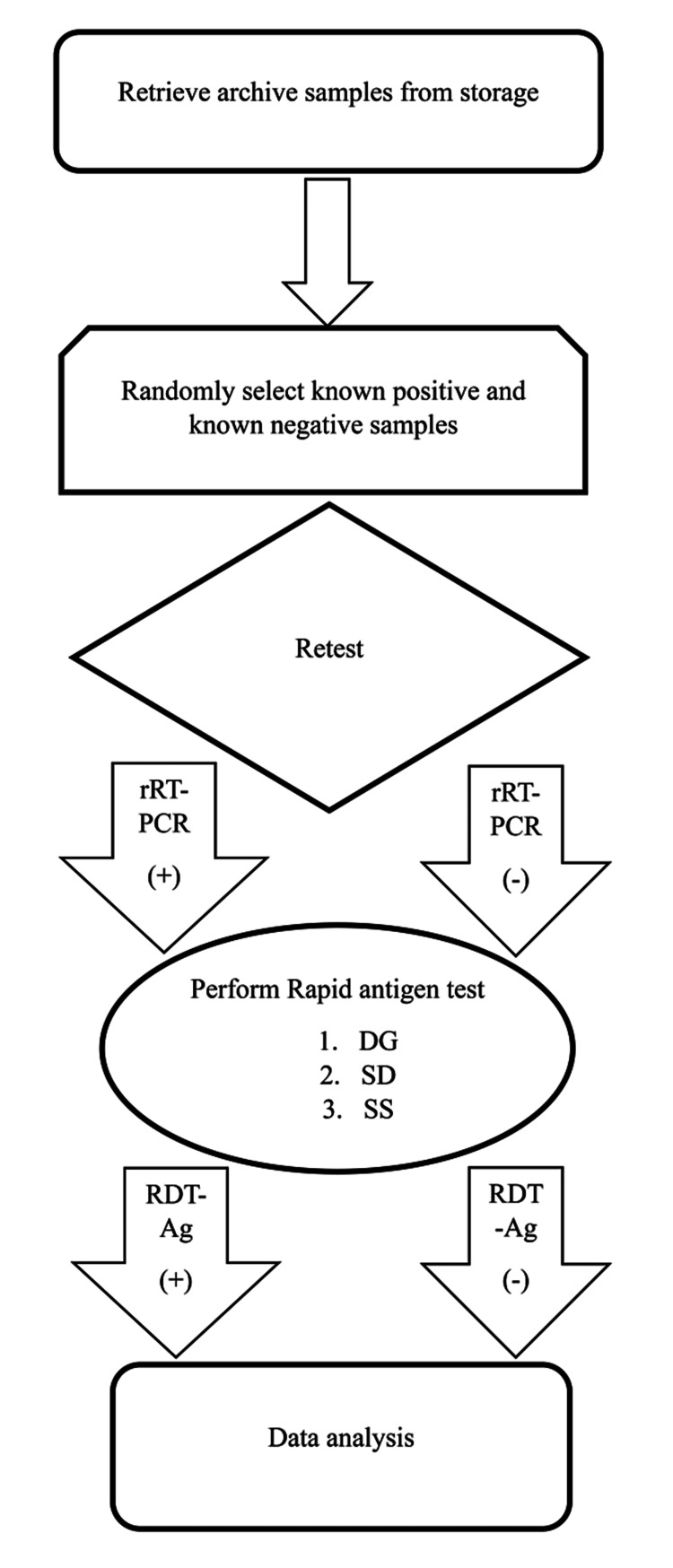
Flow chart depicting archival sample selection

## Appendices

Figure 1 shows the flow chart illustrating archival sample selection.

Working aid for COVID-19 testing using Sansure reagents on the ma6000

A.      Reagents

1.       Sample release reagent

2.       Sample storage buffer

3.       Swabs

4.       Nucleic acid amplification kit

B.      Consumables

1.       Strips of 0.2 mL PCR tubes

2.       Strip caps

3.       1.5 mL Eppendorf tubes

4.       Cryostorage box

5.       Ice chest

6.       Ice packs

C.      Sample collection

1.       With the swab, collect specimens from either the oropharynx or nasopharynx.

2.       Dip the tip of the swab in the sample storage buffer and cut off the excess shaft of the swab.

3.       Cap the tube tightly and stick it with a barcode label.

D.      Sample transport

1.       Pack all labeled samples in the cryostorage box.

2.       Cover the cryostorage box, put it in a biohazard bag, and finally place it in an ice chest with ice packs.

3.       Disinfect the ice chest and carry it with bare hands upstairs for sample processing.

E.       Sample processing

Sample preparatory room

1.       Check the order of samples received on the bench.

2.       Vortex each sample for about 10 seconds.

3.       Spin samples down briefly in the bench-top centrifuge

4.       Transfer samples onto a rack and keep samples in the biosafety cabinet.

5.       Mount the appropriate numbers of the 0.2 mL PCR tubes based on the number of samples to be processed; Add two extra tubes to be used for the processing of controls (1 negative and 1 positive).

6.       Aliquot 10 uL of sample release reagent into each of the 0.2 mL PCR tubes.

7.       To each of the tubes, add 10 uL of a specific sample; ensure to mix during the dispensation of the sample into the reagent and incubate for 10 minutes. Whilst waiting, proceed to prepare the master-mix.

Master-mix preparatory room

1.       Using the microfuge, spin the reagents in the nucleic acid amplification briefly.

2.       Keep all the tubes containing the reagents on a rack and place them in the laminar flow cabinet. Ensure to open all reagents in the laminar flow cabinet.

3.       Prepare the master-mix for N samples using the contents of the nucleic acid kit. Make sure to add all reagents into a 1.5 mL Eppendorf tube. Refer to Table 6 below for details

**Table 6:** Master-mix preparation PCR: polymerase chain reaction

Reagent	X 1 uL	X (N+1) uL
PCR mix	26	-
PCR enzyme mix	4	-
Total	30	-

Master-mix-template addition in sample preparatory room

1.       Export the master-mix in the Eppendorf tube on a rack placed at the window between the sample preparatory and master-mix rooms.

2.       After the 10 minutes of incubation of samples and the sample release reagent, dispense 30 uL of the master-mix into each tube that has a sample.

3.       Export the 0.2mL tubes through the window into the PCR room.

4.       Using the microfuge, spin strips of 0.2 mL tubes down briefly before placing in the PCR and starting the test.

Setting up test on the PCR device

Refer to the procedures documented in the operational quality report.

Analysis of test results

1.       The test identifies 3 targets:

a.       FAM- SARS-CoV-2 ORF 1ab

b.       ROX- SARS-CoV-2 N gene

c.       CY5- Human-specific target acting as the internal control.

2.       For a valid test, the positive control of the setup should have sigmoidal curves with Ct values for all 3 targets; the negative control is not expected to show any sigmoidal curve, but if it does, it should record a Ct value >40.

3.       For each sample tested, whether negative or positive, the internal control Ct detected at the CY5 channel should record a Ct<40 to be valid; If 40 or above, then the sample must be retaken from the patient and tested again.

4.       For a positive sample, in addition to the presence of the internal control (i.e., Ct<40), at least the FAM or ROX or both must show sigmoidal curves with Ct values <40.

S1: Immunochromatographic (ICT) assay principle and procedure

Assay principle

The three test kits - DG Rapid, SD Rapid, and SS Rapid - operate on the principle of immunochromatographic assays.

The principle of immunochromatography involves the separation of components in a mixture through a medium using capillary force and the specific and rapid binding of an antibody to its antigen. In these assays, each cassette is a dry medium that has been coated separately with an anti-coronavirus (anti-nucleocapsid) antibody molecule; this is a monoclonal antibody directed against nucleocapsid protein of 2019-nCoV and goat anti-chicken IgY antibody (control line). Two free colloidal gold labeled antibodies, i.e. anti-nucleocapsid antibody as well as chicken IgY, will be sprayed on the conjugate pad.

Once the nasopharyngeal swab specimen is collected and diluted in extraction buffer, this diluted specimen will be applied on the sample pad. The specimen in the buffer will pass through the conjugate pad and bind with an anti-nucleocapsid antibody conjugated with colloidal gold on the conjugate pad and will form a complex of antigen-antibody colloidal gold and will migrate towards test and control lines. Thus, the formed complex of antigen-antibody-colloidal gold will migrate through capillary action and bind with the coated antibody molecules at the test line, thus providing a reactive result.

If there is no formation of antigen-antibody colloidal gold complex, it will not bind with the test line and there will not be any development of test lines. Chicken IgY conjugated with colloidal gold will bind with control line antibody irrespective of reactive/non-reactive specimens.

Immunochromatographic analysis procedure

1.      Archived rRT-PCR positive and rRT-PCR negative COVID-19 respiratory samples, mainly nasopharyngeal swabs were sampled randomly.

2.      Three different brands of COVID-19 test kits were sampled from major distributors of laboratory diagnostic materials.

3.      The three antigen kits consist of qualitative membrane-based immunoassays based on the detection of the SARS CoV-2 nucleocapsid antigen for the rapid detection of COVID-19-positive samples.

4.      The swabs from the different kits were incubated at room temperature for 15 minutes in the archived viral transport medium previously analyzed in rRT-PCR. This incubation step is to allow time for the swab to get soaked with the sample and to mimic in real life the patient’s nasopharyngeal swab.

5.      All tests were performed according to the manufacturer’s instructions.

6.      A test is considered valid if the control line is visible, considered positive if the test line and the control line are visible, and negative if only the control line is visible.

7.      The archived specimen was picked at random before testing and all assays were performed by one operator on each test kit brand and read by two different operators blinded to each other’s results. In cases of discrepancy, a third operator reading was requested to decide the outcome of the result, especially with faint-colored test lines.
